# Beyond Bell’s theorem: realism and locality without Bell-type correlations

**DOI:** 10.1038/s41598-017-14956-y

**Published:** 2017-11-06

**Authors:** F. De Zela

**Affiliations:** Departamento de Ciencias, Sección Física, Pontificia Universidad Católica del Perú, Apartado 1761, Lima, Peru

## Abstract

The long-lasting view of entanglement as the characteristic trait of quantum mechanics has been recently challenged by experimental demonstrations of non-quantum entanglement. This motivates a review of the meaning of Bell violations, which have been widely taken to prove the impossibility of a realistic interpretation of quantum mechanics and as a manifestation of its non-local character. This work provides new theoretical evidence for the need of reviewing the meaning of Bell violations, especially when they occur outside the quantum framework. We present a local-realistic model that reproduces quantum predictions concerning Bell tests. We claim that local-realism is fully compatible with correlations that are not of the Bell type and therefore lie outside the scope of Bell’s theorem. Most experimental Bell tests involve either spin vectors spanning the Bloch sphere or Stokes vectors spanning the Poincaré sphere. A suitable statistical tool that allows assessing correlations between vectors is given by inner-product-type correlations. Using them, it is possible to reproduce quantum predictions for all Bell states, thereby explaining experimental results of Bell tests within a local-realistic framework.

## Introduction

Bell inequalities were originally derived to show the impossibility of “completing” quantum mechanics by means of a local realistic model^1^. In view of several experimental tests that violate Bell inequalities, it has been widely accepted that realism and locality cannot hold together. In recent times, however, such a conclusion has been challenged, because entanglement – a key feature of Bell violations – could be exhibited at the classical level^2–10^, and this led people to produce “non-quantum Bell violations”. From a practical point of view, these violations can be variously used: as an entanglement witness^11^, as a tool to detect hidden coherences^3,7,12–16^, as a means to simulate quantum information processing^17–19^, etc. From a foundational point of view, a key question remains open: How can a local-realistic model and its experimental implementation lead to a Bell violation? The present work addresses this question and explores the possibility that Bell violations do not prove wrong the conjunction of realism and locality alone, but the conjunction consisting of realism, locality *and* Bell-type correlations. These correlations were a natural choice from Bell’s original viewpoint, in which binary outputs played a central role. Indeed, if we consider the standard array in which Stern-Gerlach-type measurements are performed by two parties – “Alice” and “Bob” – at two distant sites, then at each site we have a binary output. Bell-type correlations were constructed so as to capture this binary nature of the output. They were also proved to be constrained by so-called Bell-type inequalities^20,21^, which may be violated by quantum correlations. Now, whereas realism and locality are necessary assumptions to establish Bell-type correlations, these correlations are not the only possible choice that is compatible with realism and locality. In other words, realism and locality are necessary but not sufficient conditions for deriving Bell-type inequalities. This fact is well known and has been repeatedly stressed in the past; but emphasis has been placed on more technical, additional assumptions, such as the “fair-sampling assumption”, “free will”, “non-signaling”, etc.^22,23^. Here we want to focus on the assumption that correlations must be of the Bell type. As we shall see, other type of correlations that are fully compatible with realism and locality can be used to address the task of explaining experimental observations. The correlations we will address have been widely used in classical, stochastic electrodynamics and also in standard statistics^24,25^. In Bell tests, Alice and Bob’s measurement outcomes are eventually encoded in terms of vectors, e.g., vectors on the Bloch sphere in the case of spin-1/2 particles, and vectors on the Poincaré sphere in the case of polarized photons. Generally, correlations between vectors are not properly described by quantities such as Bell-type correlations, which are constructed in terms of scalar quantities alone. Inner-product, Pearson-type correlations could be a more appropriate tool for this case. Our aim is to see how far we can go with a local-realistic, classical approach, by paralleling the quantum approach. This should help us assessing how much an approach departs from the other, thereby contributing to fix the yet undetermined quantum-classical border. A key question thus concerns the definition of statistical correlations by means of inner products. Is this definition allowed only within the quantum framework? This point will be discussed with the help of Gudder’s theorem^26^. To this end, we first discuss the relevant background, with special emphasis on the derivation of Born’s rule from Gudder’s theorem. As well known, Born’s rule is at the basis of quantum correlations. We will thus identify all quantum correlations that are relevant to standard Bell tests. These are the ones that should be reproduced by a local-realistic model. Such a model is presented and, finally, we discuss our results.

## Bell-type and quantum correlations

### Standard Bell tests

As is well known, standard Bell tests involve two types of correlations: Bell-type correlations 〈*AB*〉_*Bell*_ and quantum correlations 〈*AB*〉_*QM*_. Representative examples of these are given by

1 $${\langle AB\rangle }_{Bell}=\int \rho (\lambda )A(\lambda )B(\lambda )d\lambda ,$$

2 $${\langle AB\rangle }_{QM}=\langle {\boldsymbol{\Psi }}|(\hat{{\boldsymbol{a}}}\cdot {{\boldsymbol{\sigma }}}^{A})\,(\hat{{\boldsymbol{b}}}\cdot {{\boldsymbol{\sigma }}}^{B})|{\boldsymbol{\Psi }}\rangle \mathrm{.}$$

Unit vectors $\hat{{\boldsymbol{a}}}$ and $\hat{{\boldsymbol{b}}}$ span the Bloch sphere in the case of spin-1/2 particles and the Poincaré sphere in the case of polarized photons. These unit vectors can also be thought of as fixing the respective orientations of Stern-Gerlach apparatuses for spin measurements, or the corresponding setups for measuring Stokes vectors in the optical case. In the quantum case, $\hat{{\boldsymbol{a}}}$ and $\hat{{\boldsymbol{b}}}$ also fix the observables being measured by Alice and Bob: $\hat{{\boldsymbol{a}}}\cdot {{\boldsymbol{\sigma }}}^{A}$ and $\hat{{\boldsymbol{b}}}\cdot {{\boldsymbol{\sigma }}}^{B}$, respectively. These observables produce a binary output that is encoded by *A*(*λ*) and *B*(*λ*) in the local-realistic case, in which “hidden variables” *λ* are assumed to determine whether the output is +1 or −1 in each case, i.e., *A*(*λ*) = ±1 and *B*(*λ*) = ±1. The probability distribution of *λ* is given by *ρ*(*λ*) ≥ 0, with ∫*ρ*(*λ*)*dλ* = 1.

Various, so-called Bell measures can be constructed in terms of the above correlations. The Bell-Clauser-Horne-Shimony-Holt (Bell-CHSH) measure is a particularly suitable one for experimental tests^20^. It is given by

3 $$S=\langle AB\rangle +\langle AB^{\prime} \rangle +\langle A^{\prime} B\rangle -\langle A^{\prime} B^{\prime} \rangle .$$

Here, *A* and *A*′ refer to two different settings: $\hat{{\boldsymbol{a}}}$ and $\hat{{\boldsymbol{a}}}^{\prime}$, respectively, and similarly for *B*, *B*′. In the case of 〈*AB*〉_*Bell*_, on using the identity (*A*(*λ*) + *A*′(*λ*))*B*(*λ*) + (*A*(*λ*) − *A*′(*λ*))*B*(*λ*) = ±2 together with |∫*f*(*λ*)*dλ*| ≤ ∫|*f*(*λ*)|*dλ*, one can readily prove that |*S*| ≤ 2 for any choice of states and observables, while one can get |*S*| = 2$\sqrt{2}$ for 〈*AB*〉_*QM*_, under appropriate choice of states and observables^27,28^. The fact that |*S*| > 2 for 〈*AB*〉_*QM*_ has led people to conclude that quantum correlations can be “stronger” than classical, Bell-type ones. Bell violations of constraints such as |*S*| ≤2 have also prompted the conclusion that quantum mechanics is non-local.

Turning now to 〈*AB*〉_*Bell*_, we should stress that it is a local quantity, in spite of involving two distant settings. Indeed, it derives from

4 $${\langle AB\rangle }_{Bell}=\sum _{\alpha ,\beta =\pm 1}\alpha \beta p(\alpha ,\,\beta |\hat{{\boldsymbol{a}}},\,\hat{{\boldsymbol{b}}}),$$

with $p(\alpha ,\,\beta |\hat{{\boldsymbol{a}}},\,\hat{{\boldsymbol{b}}})={\int }_{\Lambda }\rho (\lambda )p(\alpha ,\,\beta |\hat{{\boldsymbol{a}}},\,\hat{{\boldsymbol{b}}},\,\lambda )d\lambda$ being the conditional probability of *α* = ±1 and *β* = ±1 occurring when measuring settings ($(\hat{{\boldsymbol{a}}},\,\hat{{\boldsymbol{b}}})$) are fixed at distant locations, while shared randomness is fixed by the hidden-variable *λ*. On invoking the locality assumption, one sets

5 $$p(\alpha ,\,\beta |\hat{{\boldsymbol{a}}},\,\hat{{\boldsymbol{b}}},\,\lambda )=p(\alpha |\hat{{\boldsymbol{a}}},\,\lambda )p(\beta |\hat{{\boldsymbol{b}}},\,\lambda ),$$

thereby getting Eq. (1) from (4).

Modulo some loopholes, experiments have shown that we must resort to correlations such as 〈*AB*〉_*QM*_ rather than 〈*AB*〉_*Bell*_, in order to describe experimental findings. The theoretical axiomatic behind 〈*AB*〉_*QM*_ is that of quantum mechanics, more specifically Born’s rule. The theoretical axiomatic behind 〈*AB*〉_*Bell*_ is Kolmogorov’s axiomatic of probability theory^29^. We could thus be tempted to conclude that Kolmogorov’s axiomatic is in conflict with quantum mechanics. Gleason’s attempt^30^ to derive Born’s rule from Kolmogorov’s axiomatic might be seen as an attempt to show that quantum mechanics is not in conflict with our most basic notions of probability. However, Gleason’s derivation of Born’s rule excluded two-dimensional Hilbert spaces, which are of paramount importance in quantum mechanics. The 2*D* case was afterwards covered by Busch’s approach^31^, but at the cost of conflicting Kolmogorov’s axiomatic. A third attempt was presented recently^32–34^, in which the 2*D* case is covered without conflicting Kolmogorov’s axiomatic. Moreover, this axiomatic appears as a special case of what can be seen as the most basic physical concept, namely the concept of a signed measure. Our basic notion of a measure is encoded by a non-negative function *m* over a *σ*-algebra, a function that is required to satisfy *m*(*A* ∪ *B*) = *m*(*A*) + *m*(*B*), whenever *A* ∩ *B* = ∅. A measure essentially quantifies how many times a given unit fits into what is being measured. Very often, it is convenient to provide this measure with a sign, thereby defining a signed measure. This allows us to count not only how many times the unit fits into something, but also how many times the unit is missing when trying to reach some given amount. A probability measure is a non-negative measure. As such, it is a special case of a signed measure. Gudder’s theorem allows us to deal with signed measures that lead to the Born rule when properly restricted by physical requirements. Moreover, we can deal with correlations between measured quantities and be in accordance with experimental outcomes of Bell tests, without invoking any quantum concept. We next dwell extensively on these issues.

### Classical and quantum probabilities

Eq. (2) is based on Born’s rule, which in its simplest form establishes that |〈*ϕ*|*ψ*〉|^2^ is the probability of finding a system in state |*ϕ*〉, when it was previously prepared in state |*ψ*〉. We should stress that Born’s formula applies irrespective of location. That is, it works the same when we have, say, |〈*ϕ*(***x***)|*ψ*(***y***)〉|^2^, with ***x*** ≠ ***y***. This is because Born’s formula is just a measure for the overlap of two vectors, |*ϕ*〉 and |*ψ*〉, irrespective of any positional degrees of freedom that we may attach to them. Hence, a potential built-in non-locality is already there. It is thus no wonder that non-locality shows up in related expressions, such as the probability rules that we interpret as “correlations”.

We can make more obvious that Born’s formula requires only a vectorial scalar product – rather than the square of it – by writing this formula in the form $Tr({\rho }_{\varphi }^{\dagger }{\rho }_{\psi })$, where *ρ*_*ϕ*_ = |*ϕ*〉〈*ϕ*| and *ρ*_*ψ*_ = |*ψ*〉〈*ψ*|. Here, we recognize the Hilbert-Schmidt inner product for operators *A* and *B*, namely *Tr*(*A* ^†^ *B*). The latter is just a reshaping of the standard, Euclidean inner product between vectors: ***a*** ⋅ ***b*** = ∑_*i*_ *a*_*i*_ *b*_*i*_, which follows from “vectorializing” the matrix representations of *A* and *B*. By “vectorializing” a matrix we mean writing its rows as column vectors, one on top of the next. It is thus possible that Born’s rule by itself does not convey any feature that must be dubbed a “quantum mechanical” one. In order to elucidate this issue, we focus on two-level systems (qubits), the ones most relevant for measurements having a binary output, i.e., those entering the Bell tests we address here.

As we shall see, the correlations entering Bell tests may be expressed as inner products. This is just a particular instance of a general statement regarding physical measurements. As we said before, measurements essentially consist on counting how many times some given unit fits into the observable being measured, and the mathematical tool that captures this basic notion is a non-negative map *m* over the elements of some set (a *σ*-algebra). This map is required to satisfy *m*(*A*∪*B*) = *m*(*A*) + *m*(*B*), whenever *A*∩*B* = ∅. A particular case of a measure is the “probability measure” of quantum mechanics, which is defined over the projection lattice ${\mathscr{P}}( {\mathcal H} )$ of Hilbert space ${\mathcal H}$. In this case, one requires for ${P}_{i},\,{P}_{j}\in {\mathscr{P}}( {\mathcal H} )$ that *m*(*P*_*i*_ + *P*_*j*_) = *m*(*P*_*i*_) + *m*(*P*_*j*_), whenever *P*_*i*_ *P*_*j*_ = 0. We can relax the requirement that *m* be non-negative, in order to include those cases in which we find it convenient to add a sign to our measurement results. In such a case, the above definition of a measure may be replaced by another one that is the subject matter of Gudder’s theorem^26^. This theorem addresses an inner product vector space *V* and a continuous function *f* that is orthogonally additive and maps vectors *r* ∈ *V* to the reals. Such a function can be seen as a sign-carrying measure. The definition of orthogonal additivity reads as follows.

**Definition 2.1**.

6 $$f:\,V\to {\mathbb{R}}\,is\,orthogonally\,additive,\,if\quad f(r+r^{\prime} )=f(r)+f(r^{\prime} )\quad whenever\quad r\cdot r^{\prime} =0.$$

In terms of the foregoing definition, Gudder proved^26^ the following result:

#### **Theorem 2.1.**

If $f:\,V\to {\mathbb{R}}$ *is orthogonally additive and continuous, then it is of the form*

7 $$f(r)=c(r\cdot r)+k\cdot r,$$

where $$c\in {\mathbb{R}}$$ and *k* ∈ *V*.

Let us now focus on the space *V*_4_, with elements ${\boldsymbol{ {\mathcal R} }}=({r}_{0},{r}_{1},{r}_{2},{r}_{3})\equiv ({r}_{0},{\bf{r}})$ that we can put in one-to-one correspondence with qubits |*ψ*〉〈*ψ*|:

8 $${R}_{\psi }\equiv |\psi \rangle \langle \psi |=\frac{1}{2}\sum _{\mu =0}^{3}{r}_{\mu }{\sigma }_{\mu },$$

where *σ*_0_ is the identity and *σ*_*μ*_ = _1,2,3_ the Pauli matrices.

We want to define a sign-carrying measure *f*_*ϕ*_ relative to a given qubit $|\varphi \rangle \langle \varphi |\leftrightarrow {{\boldsymbol{ {\mathcal R} }}}_{\varphi }\equiv \mathrm{(1,}\,{\hat{{\bf{n}}}}_{\varphi })$. Function *f*_*ϕ*_ is assumed to satisfy the requirements of Theorem 2.1 and the following, additional ones:

1. ${f}_{\varphi }({{\boldsymbol{ {\mathcal R} }}}_{\varphi })=1$, which means that our unit of measure fits exactly one time into itself.
2. ${f}_{\varphi }({{\boldsymbol{ {\mathcal R} }}}_{{\varphi }_{\perp }})={f}_{\varphi }({{\boldsymbol{ {\mathcal R} }}}_{{\varphi }_{\perp }^{^{\prime} }})=0$ for vectors ${{\boldsymbol{ {\mathcal R} }}}_{{\varphi }_{\perp }}\equiv \mathrm{(1,}\,-{\hat{{\bf{n}}}}_{\varphi })$ and ${{\boldsymbol{ {\mathcal R} }}}_{{\varphi }_{\perp }^{^{\prime} }}\equiv (-\mathrm{1,}\,{\hat{{\bf{n}}}}_{\varphi })$ that are orthogonal to ${{\boldsymbol{ {\mathcal R} }}}_{\varphi }$.
3. ${f}_{\varphi }[\mathrm{(1,}\,{\hat{{\bf{n}}}}_{{\psi }})]\in [\mathrm{0,}\,1]$ for any four-vector ${{\boldsymbol{ {\mathcal R} }}}_{\psi }=\mathrm{(1,}\,{\hat{{\bf{n}}}}_{\psi })\leftrightarrow |\psi \rangle \langle \psi |\equiv {P}_{\psi }$. This last requirement allows us to use *f*_*ϕ*_ as a probability measure, when applied to projectors ${P}_{\psi }\in {\mathscr{P}}({ {\mathcal H} }_{2})$.

On applying Gudder’s theorem one readily obtains^32–34^

9 $${f}_{\varphi }({\boldsymbol{ {\mathcal R} }})=\frac{1}{2}\mathrm{(1,}\,{\hat{{\bf{n}}}}_{\varphi })\cdot ({r}_{0},\,{\bf{r}})=\frac{1}{2}({r}_{0}+{\hat{{\bf{n}}}}_{\varphi }\cdot {\bf{r}})\mathrm{.}$$

Thus, under the above conditions ${f}_{\varphi }({\boldsymbol{ {\mathcal R} }})$ turns out to be an inner product. It can be specified either in vector space *V*_4_, where it is given by the Euclidean inner product, or in the space of linear operators acting on the two-dimensional Hilbert space ${ {\mathcal H} }_{2}$, where it is given by the Hilbert-Schmidt inner product *Tr*(*A* ^†^ *B*). Indeed, on view of ${\boldsymbol{ {\mathcal R} }}\leftrightarrow {R}_{\psi }\equiv {\rho }_{\psi }={\sum }_{\mu }\,{r}_{\mu }{\sigma }_{\mu }\mathrm{/2}$, see Eq. (8), and $\mathrm{(1,}\,{\hat{{\bf{n}}}}_{\varphi })\leftrightarrow {P}_{\varphi }\equiv {\rho }_{\varphi }=(I+{\hat{{\bf{n}}}}_{\varphi }\cdot {\boldsymbol{\sigma }})\mathrm{/2}$, we can also write

10 $${f}_{\varphi }({\boldsymbol{ {\mathcal R} }})=Tr({\rho }_{\varphi }^{\dagger }{\rho }_{\psi }\mathrm{).}$$

Generally, ${f}_{\varphi }({\boldsymbol{ {\mathcal R} }})\in [-\mathrm{1,}\,1]$; but if we restrict ourselves to apply *f*_*ϕ*_(*r*) on vectors $\mathrm{(1,}\,{\hat{{\bf{n}}}}_{\psi })\in {V}_{4}$, then ${f}_{\varphi }\mathrm{[(1,}\,{\hat{{\bf{n}}}}_{\psi })]\in [\mathrm{0,}\,1]$ and we may use *f*_*ϕ*_ as a probability measure. This is the measure entering Born’s rule, which was first derived from a somewhat different axiomatic in Gleason’s theorem^30^:

11 $${f}_{\varphi }({P}_{\psi })=|\langle \varphi |\psi \rangle {|}^{2}=Tr({P}_{\varphi }{P}_{\psi })\mathrm{.}$$

In summary, a sign-carrying measure is the fundamental concept behind our quantification of correlations between vectors. This measure turns out to be an inner product. By itself, it does not contain information about any possible cause-effect relationship between the involved vectors. In order to pinpoint such a relationship, we should introduce the additional notion of “transport”, so that the two vectors can be brought to one and the same point of the underlying manifold where they are defined, be it the space-time manifold or any other one.

It is worthwhile to stress that correlations between vectors are a particular instance of correlations between n-tuples. An example of the latter is Pearson’s correlation^35^, which is used when one aims at comparing pairs of events (*x*_*i*_, *y*_*i*_), in order to assess whether they are correlated or not. These events often occur at two different locations, where they are repeatedly registered. One then constructs n-tuples (*x*_1_,*x*_2_, …, *x*_*n*_) and (*y*_1_,*y*_2_, …, *y*_*n*_) and defines the correlation

12 $${C}_{P}=\frac{{\sum }_{i}({x}_{i}-\overline{x})({y}_{i}-\overline{y})}{\sqrt{{\sum }_{j}{({x}_{j}-\overline{x})}^{2}}\sqrt{{\sum }_{j}{({y}_{j}-\overline{y})}^{2}}},$$

which can be written in terms of unit-normalized n-tuples as

13 $${C}_{P}=\hat{{\bf{x}}}\cdot \hat{{\bf{y}}}.$$

It is a matter of convention to define *C*_*P*_ ∈ [−1, 1], as it has been defined in Eq. (12). It is indeed up to us to decide whether some given metric is useful or not. The same holds true for correlations such as 〈*AB*〉_*Bell*_ and 〈*AB*〉_*QM*_, cf. Eqs (1) and (2). For the sake of comparing quantum and classical correlations, we recall next some quantum correlations that are relevant to Bell tests.

### Quantum correlations that are relevant to Bell tests

As already remarked, the quantum correlation 〈*AB*〉_*QM*_, see Eq. (2), stems from Born’s rule: Tr(Π*ρ*), where Π refers here to a projector and *ρ* to the density matrix representing the state that is subjected to measurement. In a standard Bell test, one deals with projective measurements performed by two parties. The mathematics we use in this case does not encode a unique physical picture. This is because (quantum) correlations reproducing our measurements only refer to two observables. We may imagine them as being related to two distant parties, or else we can relate these two observables to one and the same physical object, e.g., a classical light beam. In such cases, the appropriate projectors are of the form, say, ${\Pi }^{A}(\hat{{\boldsymbol{a}}},\,+)\otimes {\Pi }^{B}(\hat{{\boldsymbol{b}}},\,-)$, and so on, while the states being considered are of the form *ρ* = |Φ〉〈Φ|, with $|{\rm{\Phi }}\rangle \in { {\mathcal H} }_{A}\otimes { {\mathcal H} }_{B}$. Here,

14 $${{\rm{\Pi }}}^{A}(\hat{{\boldsymbol{a}}},\,\alpha =\pm \mathrm{1)}=\frac{1}{2}({{\mathbb{I}}}^{A}+\alpha \hat{{\boldsymbol{a}}}\cdot {{\boldsymbol{\sigma }}}^{A}),$$

and similarly for ${{\rm{\Pi }}}^{B}(\hat{{\boldsymbol{b}}},\beta =\pm \mathrm{1)}$. As already said, in most Bell tests unit vectors $\hat{{\boldsymbol{a}}}$ and $\hat{{\boldsymbol{b}}}$ specify directions on the Bloch or on the Poincaré sphere. These directions are in turn fixed by the experimental arrangement that performs the projective measurements. Let us denote by ${P}_{\hat{{\boldsymbol{a}}},\hat{{\boldsymbol{b}}}}(\alpha ,\,\beta )$ the probability to obtain *α* = ±1 and *β* = ±1 as a result of measuring qubit *A* and qubit *B* along directions $\hat{{\boldsymbol{a}}}$ and $\hat{{\boldsymbol{b}}}$, respectively. Born’s rule states that

15 $$\begin{array}{c}{P}_{\hat{{\boldsymbol{a}}},\hat{{\boldsymbol{b}}}}(\alpha ,\,\beta )={\langle {{\rm{\Pi }}}^{A}(\hat{{\boldsymbol{a}}},\alpha )\otimes {{\rm{\Pi }}}^{B}(\hat{{\boldsymbol{b}}},\beta )\rangle }_{{\rm{\Phi }}}\\ \quad \quad \quad \quad \,=\,\frac{1}{4}{\langle {{\mathbb{I}}}^{AB}+\alpha \hat{{\boldsymbol{a}}}\cdot {{\boldsymbol{\sigma }}}^{A}\otimes {{\mathbb{I}}}^{B}+\beta {{\mathbb{I}}}^{A}\otimes \hat{{\boldsymbol{b}}}\cdot {{\boldsymbol{\sigma }}}^{B}+\alpha \beta \hat{{\boldsymbol{a}}}\cdot {{\boldsymbol{\sigma }}}^{A}\otimes \hat{{\boldsymbol{b}}}\cdot {{\boldsymbol{\sigma }}}^{B}\rangle }_{{\rm{\Phi }}}\mathrm{.}\end{array}$$

We can then obtain the quantum correlation 〈*AB*〉_*QM*_, see Eq. (2), as:

16 $${\langle AB\rangle }_{QM}={C}_{QM}(\hat{{\boldsymbol{a}}},\,\hat{{\boldsymbol{b}}})\equiv \sum _{\alpha ,\,\beta =\pm 1}\alpha \beta {P}_{\hat{{\boldsymbol{a}}},\hat{{\boldsymbol{b}}}}(\alpha ,\,\beta )={\langle (\hat{{\boldsymbol{a}}}\cdot {{\boldsymbol{\sigma }}}^{A})\otimes (\hat{{\boldsymbol{b}}}\cdot {{\boldsymbol{\sigma }}}^{B})\rangle }_{{\rm{\Phi }}}.$$

These correlations are the ones used in Bell tests, i.e., they can violate Bell-type inequalities. However, experimental outputs are not directly given by correlations such as 〈*AB*〉_*QM*_, but by correlations of the type ${P}_{\hat{{\boldsymbol{a}}},\hat{{\boldsymbol{b}}}}(\alpha ,\,\beta )$, while relevant states in Bell tests are, e.g., maximally entangled states such as the Bell states, for which $\langle \hat{{\boldsymbol{a}}}\cdot {{\boldsymbol{\sigma }}}^{A}\otimes {{\mathbb{I}}}^{B}\rangle =\langle {{\mathbb{I}}}^{A}\otimes \hat{{\boldsymbol{b}}}\cdot {{\boldsymbol{\sigma }}}^{B}\rangle =0$. Thus,

17 $${P}_{\hat{{\boldsymbol{a}}},\hat{{\boldsymbol{b}}}}(\alpha ,\,\beta )=\frac{1}{4}[1+\alpha \beta {\langle (\hat{{\boldsymbol{a}}}\cdot {{\boldsymbol{\sigma }}}^{A})\otimes (\hat{{\boldsymbol{b}}}\cdot {{\boldsymbol{\sigma }}}^{B})\rangle }_{{{\rm{\Phi }}}_{Bell}}]\mathrm{.}$$

Our next task is to check whether it is possible to reproduce quantum predictions of the above type within a local-realistic framework. To this end, we first make precise what these quantum predictions are in the case of Bell states.

## Bell violations

For the sake of concreteness, we shall refer to an optical CHSH test being performed with horizontally (H) and vertically (V) polarized, classical light beams. The same test can be performed with horizontally and vertically polarized photons. Bell states are given by |Φ^±^〉 = (|*H*〉|*H*〉 ± |*V*〉|*V*〉)/$\sqrt{2}$ and |Ψ^±^〉 = (|*H*〉|*V*〉 ± |*V*〉|*H*〉)/$\sqrt{2}$. The fact that we use Dirac notation does not mean that we are attaching any quantum feature to these states. They can be thought of as spinors or as Jones vectors^24^. The quantum expressions for the relevant correlations read

18a $$\langle {{\rm{\Phi }}}^{+}|(\hat{{\boldsymbol{a}}}\cdot {{\boldsymbol{\sigma }}}^{A})\otimes (\hat{{\boldsymbol{b}}}\cdot {{\boldsymbol{\sigma }}}^{B})|{{\rm{\Phi }}}^{+}\rangle ={a}_{1}{b}_{1}-{a}_{2}{b}_{2}+{a}_{3}{b}_{3}$$

18b $$\langle {{\rm{\Phi }}}^{-}|(\hat{{\boldsymbol{a}}}\cdot {{\boldsymbol{\sigma }}}^{A})\otimes (\hat{{\boldsymbol{b}}}\cdot {{\boldsymbol{\sigma }}}^{B})|{{\rm{\Phi }}}^{-}\rangle =-{a}_{1}{b}_{1}+{a}_{2}{b}_{2}+{a}_{3}{b}_{3}$$

18c $$\langle {{\rm{\Psi }}}^{+}|(\hat{{\boldsymbol{a}}}\cdot {{\boldsymbol{\sigma }}}^{A})\otimes (\hat{{\boldsymbol{b}}}\cdot {{\boldsymbol{\sigma }}}^{B})|{{\rm{\Psi }}}^{+}\rangle ={a}_{1}{b}_{1}+{a}_{2}{b}_{2}-{a}_{3}{b}_{3}$$

18d $$\langle {{\rm{\Psi }}}^{-}|(\hat{{\boldsymbol{a}}}\cdot {{\boldsymbol{\sigma }}}^{A})\otimes (\hat{{\boldsymbol{b}}}\cdot {{\boldsymbol{\sigma }}}^{B})|{{\rm{\Psi }}}^{-}\rangle =-{a}_{1}{b}_{1}-{a}_{2}{b}_{2}-{a}_{3}{b}_{3}\mathrm{.}$$

Our goal is to construct a local-realistic model that reproduces quantum expressions by starting from the following, classical correlations:

19 $${C}_{cl}(\hat{{\boldsymbol{a}}},\,\hat{{\boldsymbol{b}}})=\sum _{\alpha ,\,\beta =\pm 1}\,\alpha \beta {P}_{\hat{{\boldsymbol{a}}},\,\hat{{\boldsymbol{b}}}}^{cl}(\alpha ,\,\beta \mathrm{).}$$

Generally, we can write ${P}_{\hat{{\boldsymbol{a}}},\,\hat{{\boldsymbol{b}}}}^{cl}$ in the form^36^

20 $${P}_{\hat{{\boldsymbol{a}}},\,\hat{{\boldsymbol{b}}}}^{cl}(\alpha ,\,\beta )=\frac{1}{4}[1+\alpha {M}^{A}(\hat{{\boldsymbol{a}}},\,\hat{{\boldsymbol{b}}})+\beta {M}^{B}(\hat{{\boldsymbol{a}}},\,\hat{{\boldsymbol{b}}})+\alpha \beta {C}_{cl}(\hat{{\boldsymbol{a}}},\,\hat{{\boldsymbol{b}}})]\mathrm{.}$$

${P}_{\hat{{\boldsymbol{a}}},\,\hat{{\boldsymbol{b}}}}^{cl}(\alpha ,\,\beta )$ is thus written in terms of *C*_*cl*_ and the marginals ${M}^{A}(\hat{{\boldsymbol{a}}},\,\hat{{\boldsymbol{b}}})={\sum }_{\alpha ,\beta }\,\alpha {P}_{\hat{{\boldsymbol{a}}},\,\hat{{\boldsymbol{b}}}}^{cl}(\alpha ,\,\beta )$ and ${M}^{B}(\hat{{\boldsymbol{a}}},\,\hat{{\boldsymbol{b}}})={\sum }_{\alpha ,\beta }\,\beta {P}_{\hat{{\boldsymbol{a}}},\,\hat{{\boldsymbol{b}}}}^{cl}(\alpha ,\,\beta )$. Our model must be such that these marginals vanish, so that

21 $${P}_{\hat{{\boldsymbol{a}}},\,\hat{{\boldsymbol{b}}}}^{cl}(\alpha ,\,\beta )=\frac{1}{4}[1+\alpha \beta {C}_{cl}(\hat{{\boldsymbol{a}}},\,\hat{{\boldsymbol{b}}})],$$

with $${C}_{cl}(\hat{{\boldsymbol{a}}},\,\hat{{\boldsymbol{b}}})$$ reproducing $${C}_{QM}(\hat{{\boldsymbol{a}}},\,\hat{{\boldsymbol{b}}})$$.

The basic building blocks of our model will be operators of the form, e.g., |*ψ*〉〈*H*|, which transforms a horizontally polarized state into a conveniently chosen Jones vector |*ψ*〉. Consider for example the Jones matrix

22 $${T}_{A}({\theta }_{a},\,{\phi }_{a})=(\begin{array}{cc}{e}^{i{\phi }_{a}}\,\cos ({\theta }_{a}\mathrm{/2}) & \sin ({\theta }_{a}\mathrm{/2})\\ \sin ({\theta }_{a}\mathrm{/2}) & {e}^{i{\phi }_{a}}\,\cos ({\theta }_{a}\mathrm{/2})\end{array})\mathrm{.}$$

This is the matrix representation (in the basis {|*H*〉,|*V*〉} ≡ {(1,0)^*T*^,(0,1)^*T*^}, where *T* = transpose) of the operator *T*_*A*_(*θ*_*a*_,*φ*_*a*_) = |*ψ*_*A*1_〉〈*H*| + |*ψ*_*A*2_〉〈*V*|, with $|{\psi }_{A1}\rangle =({e}^{i{\phi }_{a}}\,\cos \,({\theta }_{a}\mathrm{/2),}\,\sin \,({\theta }_{a}{\mathrm{/2))}}^{T}$ and $|{\psi }_{A2}\rangle =(\sin ({\theta }_{a}\mathrm{/2),}{e}^{i{\phi }_{a}}\,\cos ({\theta }_{a}{\mathrm{/2))}}^{T}$, i.e., the first and second columns, respectively, of the above matrix *T*_*A*_. One can implement *T*_*A*_ and similar transformations with the help of optical elements, such as polarizing beam splitters and wave plates. The label *A* in *T*_*A*_ refers to Alice’s measurements, as explained below. As for Bob’s measurements, these will be related to the transformation

23 $${T}_{B}({\theta }_{b},\,{\phi }_{b})=(\begin{array}{cc}\cos ({\theta }_{b}\mathrm{/2}) & -\cos ({\theta }_{b}\mathrm{/2})\\ {e}^{i{\phi }_{b}}\,\sin ({\theta }_{b}\mathrm{/2}) & {e}^{i{\phi }_{b}}\,\sin ({\theta }_{b}\mathrm{/2})\end{array})\mathrm{.}$$

Our model prescribes that whenever a source produces a Bell state, the states received by Alice and Bob are those given in Table 1, up to normalization. These states contain, besides their polarization degrees of freedom, two additional ones. These are deterministic “hidden variables” that are associated to states |*X*〉, |*Y*〉, with 〈*X*|*Y*〉 = 0. Polarization measurements are insensitive to these degrees of freedom. That is, when performing polarization measurements, one effectively traces out these deterministic, binary hidden variables. Furthermore, states |*λ* ^±^〉, with 〈*λ* ^−^|*λ* ^+^〉 = 0, play the role of a stochastic hidden-variable, which takes on values *λ* ^+^ or *λ* ^−^ according to some given distribution. We will conveniently assume that each of them has a 50% chance to be produced.

**Table 1:** Alice and Bob’s inputs in correspondence with the Bell state produced by a source.

Prepared state	Alice’s input	Bob’s input
|Φ^+^〉 →	$$(|H\rangle +|V\rangle )|{X}_{A}\rangle |{\lambda }_{A}^{\pm }\rangle $$	$$(|H\rangle +|V\rangle )|{X}_{B}\rangle |{\lambda }_{B}^{\pm }\rangle $$
|Φ^−^〉 →	$$(|H\rangle -|V\rangle )|{X}_{A}\rangle |{\lambda }_{A}^{\pm }\rangle $$	$$(|H\rangle -|V\rangle )|{Y}_{B}\rangle |{\lambda }_{B}^{\pm }\rangle $$
|Ψ^+^〉 →	$$(|H\rangle +|V\rangle )|{Y}_{A}\rangle |{\lambda }_{A}^{\pm }\rangle $$	$$(|H\rangle +|V\rangle )|{X}_{B}\rangle |{\lambda }_{B}^{\pm }\rangle $$
|Ψ^−^〉 →	$$(|H\rangle -|V\rangle )|{Y}_{A}\rangle |{\lambda }_{A}^{\pm }\rangle $$	$$(|H\rangle -|V\rangle )|{Y}_{B}\rangle |{\lambda }_{B}^{\pm }\rangle $$

We further prescribe that Alice and Bob’s measurements can be described by the following transformations:

24 $$\begin{array}{c}{T}_{A}^{(tot)}=(|{\psi }_{A1}\rangle \langle H|\otimes |{X}_{A}\rangle \langle {X}_{A}|+|{\psi }_{A2}\rangle \langle V|\otimes |{Y}_{A}\rangle \langle {Y}_{A}|)\otimes |{\lambda }_{A}^{+}\rangle \langle {\lambda }_{A}^{+}|+\\ \quad \quad \,\,\,\,+(|{\psi }_{A1}^{\perp }\rangle \langle H|\otimes |{X}_{A}\rangle \langle {X}_{A}|+|{\psi }_{A2}^{\perp }\rangle \langle V|\otimes |{Y}_{A}\rangle \langle {Y}_{A}|)\otimes |{\lambda }_{A}^{-}\rangle \langle {\lambda }_{A}^{-}|\end{array}$$

and

25 $$\begin{array}{c}{T}_{B}^{(tot)}=(|{\psi }_{B1}\rangle \langle H|\otimes |{X}_{B}\rangle \langle {X}_{B}|+|{\psi }_{B2}\rangle \langle V|\otimes |{Y}_{B}\rangle \langle {Y}_{B}|)\otimes |{\lambda }_{B}^{+}\rangle \langle {\lambda }_{B}^{+}|+\\ \quad \quad \,\,\,\,+(|{\psi }_{B1}^{\perp }\rangle \langle H|\otimes |{X}_{B}\rangle \langle {X}_{B}|+|{\psi }_{B2}^{\perp }\rangle \langle V|\otimes |{Y}_{B}\rangle \langle {Y}_{B}|)\otimes |{\lambda }_{B}^{-}\rangle \langle {\lambda }_{B}^{-}\mathrm{|.}\end{array}$$

Here, $|{\psi }_{A1}^{\perp }\rangle$ and $|{\psi }_{A2}^{\perp }\rangle$ are vectors orthogonal to those in the first and second columns of the matrix *T*_*A*_, see Eq. (22), and similarly for $|{\psi }_{B1}^{\perp }\rangle$ and $|{\psi }_{B2}^{\perp }\rangle$.

Let us see, for instance, what happens when the source emits Bell states |Φ^+^〉. In case Alice receives the state corresponding to $|{\lambda }_{A}^{+}\rangle$, see Table 1, the effective polarization transformation due to ${T}_{A}^{(tot)}$ is given by |*ψ*_*A*1_〉〈*H*|, cf. Eq. (24). If she instead receives the state corresponding to $|{\lambda }_{A}^{-}\rangle$, the effective polarization transformation is given by $|{\psi }_{A1}^{\perp }\rangle \langle H|$. Thus, half of the time Alice detects the up-state |*ψ*_*A*1_〉 and half of the time the down-state $|{\psi }_{A1}^{\perp }\rangle$. “Up” and “down” correspond to the antiparallel directions that are associated to Jones vectors |*ψ*_*A*1_〉 and $|{\psi }_{A1}^{\perp }\rangle$, respectively, i.e., to antipodal points on the Poincaré sphere. Thus, the marginal ${M}^{A}(\hat{{\boldsymbol{a}}},\,\hat{{\boldsymbol{b}}})=0$, in accordance with the quantum prediction. In the same way one can see that ${M}^{B}(\hat{{\boldsymbol{a}}},\,\hat{{\boldsymbol{b}}})=0$ on Bob’s side.

Following the above prescriptions with all Bell states, the output polarization measurements of Alice and Bob read as given in Table 2.

**Table 2:** Jones vectors corresponding to Alice and Bob’s outputs.

Prepared state	Alice’s output for ${\lambda }_{A}^{+}$	Bob’s output for ${\lambda }_{B}^{+}$	Alice’s output for ${\lambda }_{A}^{-}$	Bob’s output for ${\lambda }_{B}^{-}$
|Φ^+^〉 →	$$(\begin{array}{c}{e}^{i{\phi }_{a}}\,\cos ({\theta }_{a}\mathrm{/2})\\ \sin ({\theta }_{a}\mathrm{/2})\end{array})$$	$$(\begin{array}{c}\cos ({\theta }_{b}\mathrm{/2})\\ {e}^{i{\phi }_{b}}\,\sin ({\theta }_{b}\mathrm{/2})\end{array})$$	$$(\begin{array}{c}-{e}^{i{\phi }_{a}}\,\sin ({\theta }_{a}\mathrm{/2})\\ \cos ({\theta }_{a}\mathrm{/2})\end{array})$$	$$(\begin{array}{c}-\,\sin ({\theta }_{b}\mathrm{/2})\\ {e}^{i{\phi }_{b}}\,\cos ({\theta }_{b}\mathrm{/2})\end{array})$$
|Φ^−^〉 →	$$(\begin{array}{c}{e}^{i{\phi }_{a}}\,\cos ({\theta }_{a}\mathrm{/2})\\ \sin ({\theta }_{a}\mathrm{/2})\end{array})$$	$$(\begin{array}{c}-\,\cos ({\theta }_{b}\mathrm{/2})\\ {e}^{i{\phi }_{b}}\,\sin ({\theta }_{b}\mathrm{/2})\end{array})$$	$$(\begin{array}{c}-{e}^{i{\phi }_{a}}\,\sin ({\theta }_{a}\mathrm{/2})\\ \cos ({\theta }_{a}\mathrm{/2})\end{array})$$	$$(\begin{array}{c}\sin ({\theta }_{b}\mathrm{/2})\\ {e}^{i{\phi }_{b}}\,\cos ({\theta }_{b}\mathrm{/2})\end{array})$$
|Ψ^+^〉 →	$$(\begin{array}{c}\sin ({\theta }_{a}\mathrm{/2})\\ {e}^{i{\phi }_{a}}\,\cos ({\theta }_{a}\mathrm{/2})\end{array})$$	$$(\begin{array}{c}\cos ({\theta }_{b}\mathrm{/2})\\ {e}^{i{\phi }_{b}}\,\sin ({\theta }_{b}\mathrm{/2})\end{array})$$	$$(\begin{array}{c}-\,\cos ({\theta }_{a}\mathrm{/2})\\ {e}^{i{\phi }_{a}}\,\sin ({\theta }_{a}\mathrm{/2})\end{array})$$	$$(\begin{array}{c}-\,\sin ({\theta }_{b}\mathrm{/2})\\ {e}^{i{\phi }_{b}}\,\cos ({\theta }_{b}\mathrm{/2})\end{array})$$
|Ψ^−^〉 →	$$(\begin{array}{c}\sin ({\theta }_{a}\mathrm{/2})\\ {e}^{i{\phi }_{a}}\,\cos ({\theta }_{a}\mathrm{/2})\end{array})$$	$$(\begin{array}{c}-\,\cos ({\theta }_{b}\mathrm{/2})\\ {e}^{i{\phi }_{b}}\,\sin ({\theta }_{b}\mathrm{/2})\end{array})$$	$$(\begin{array}{c}-\,\cos ({\theta }_{a}\mathrm{/2})\\ {e}^{i{\phi }_{a}}\,\sin ({\theta }_{a}\mathrm{/2})\end{array})$$	$$(\begin{array}{c}\sin ({\theta }_{b}\mathrm{/2})\\ {e}^{i{\phi }_{b}}\,\cos ({\theta }_{b}\mathrm{/2})\end{array})$$

Outputs depend on whether the random variables take the values ${\lambda }_{A/B}^{+}$ or ${\lambda }_{A/B}^{-}$.

The Stokes vector $\hat{{\boldsymbol{a}}}$ that corresponds to Alice’s output Jones vector |*ψ*_*A*_〉 is given by $\hat{{\boldsymbol{a}}}=Tr(|{\psi }_{A}\rangle \langle {\psi }_{A}|\cdot {\boldsymbol{\sigma }})$, and similarly for Bob’s Stokes vectors $\hat{{\boldsymbol{b}}}$. For example, for the Jones vectors on the first row in Table 2, i.e., the one belonging to |Φ^+^〉, we get

26 $${\hat{{a}}}_{{\lambda }_{A}^{+}}=-{\hat{{a}}}_{{\lambda }_{A}^{-}}=(\cos \,{\phi }_{a}\,\sin \,{\theta }_{a},-\sin \,{\phi }_{a}\,\sin \,{\theta }_{a},\,\cos \,{\theta }_{a}),$$

27 $${\hat{{b}}}_{{\lambda }_{B}^{+}}=-{\hat{{b}}}_{{\lambda }_{B}^{-}}=(\cos \,{\phi }_{b}\,\sin \,{\theta }_{b},\,\sin \,{\phi }_{b}\,\sin \,{\theta }_{b},\,\cos \,{\theta }_{b}\mathrm{).}$$

We want to calculate the probability ${P}_{\hat{{\bf{a}}},\hat{{\bf{b}}}}^{cl}(\alpha ,\beta )$ of Alice getting *α* = ±1 and Bob *β* = ±1, when their respective measurement devices are set to detect up/down outputs with respect to directions $\hat{{\boldsymbol{a}}}$ and $\hat{{\boldsymbol{b}}}$, respectively. At this point, we may invoke Gudder’s theorem and set ${P}_{\hat{{\bf{a}}},\hat{{\bf{b}}}}^{cl}(\alpha ,\beta )={f}_{\alpha \hat{{\bf{a}}}}(\beta \hat{{\bf{b}}})$ or else, equivalently, ${P}_{\hat{{\bf{a}}},\hat{{\bf{b}}}}^{cl}(\alpha ,\beta )={f}_{\beta \hat{{\bf{b}}}}(\alpha \hat{{\bf{a}}})$. In both cases, by applying Gudder’s theorem to Stokes vectors $\mathrm{(1,}\alpha \hat{{\bf{a}}})$ and $\mathrm{(1,}\beta \hat{{\bf{b}}})$ we get, see Eq. (9),

28 $${P}_{\hat{{\boldsymbol{a}}},\,\hat{{\boldsymbol{b}}}}^{cl}(\alpha ,\beta )=\frac{1}{4}\mathrm{(1,}\alpha \hat{{\boldsymbol{a}}})\cdot \mathrm{(1,}\beta \hat{{\boldsymbol{b}}})=\frac{1}{4}(1+\alpha \beta \hat{{\boldsymbol{a}}}\cdot \hat{{\boldsymbol{b}}})\mathrm{.}$$

Here, the normalization has been chosen so that ${\sum }_{\alpha ,\beta }{P}_{\hat{{\boldsymbol{a}}},\,\hat{{\boldsymbol{b}}}}^{cl}(\alpha ,\,\beta )=1$. Moreover, we see that the stochastic hidden variables ${\lambda }_{A/B}^{\pm }$ – which determine whether the output is “up” or “down” – are in one-to-one correspondence with *α* and *β*, according to ${\lambda }_{A}^{\pm }\leftrightarrow \alpha =\pm 1$, ${\lambda }_{B}^{\pm }\leftrightarrow \beta =\pm 1$. Indeed, by calculating the Stokes vectors that correspond to the Jones vectors of the case |Φ^+^〉, see Table 2, we obtain

29 $$\begin{array}{c}{\hat{{\boldsymbol{a}}}}_{{\lambda }_{A}^{+}}\cdot {\hat{{\boldsymbol{b}}}}_{{\lambda }_{B}^{+}}={\hat{{\boldsymbol{a}}}}_{{\lambda }_{A}^{-}}\cdot {\hat{{\boldsymbol{b}}}}_{{\lambda }_{B}^{-}}=-{\hat{{\boldsymbol{a}}}}_{{\lambda }_{A}^{+}}\cdot {\hat{{\boldsymbol{b}}}}_{{\lambda }_{B}^{-}}=-{\hat{{\boldsymbol{a}}}}_{{\lambda }_{A}^{-}}\cdot {\hat{{\boldsymbol{b}}}}_{{\lambda }_{B}^{+}}\equiv \alpha \beta \hat{{\boldsymbol{a}}}\cdot \hat{{\boldsymbol{b}}},\\ {\rm{with}}\quad \hat{{\boldsymbol{a}}}\cdot \hat{{\boldsymbol{b}}}=\,\cos \,{\theta }_{a}\,\cos \,{\theta }_{b}+\,\cos ({\phi }_{a}+{\phi }_{b})\sin \,{\theta }_{a}\,\sin \,{\theta }_{b}\mathrm{.}\end{array}$$

On replacing (29) into (31), we reproduce the quantum prediction for the Bell state |Φ^+^〉, see Eqs (17) and (18a). One can similarly show that the quantum predictions for the other three Bell states, Eqs. (18b, 18c, 18d), are also reproduced by means of the present model. Thus, our model predicts correlations ${C}_{cl}(\hat{{\boldsymbol{a}}},\,\hat{{\boldsymbol{b}}})$, see Eq. (19), that coincide with the quantum ones, ${C}_{QM}(\hat{{\boldsymbol{a}}},\,\hat{{\boldsymbol{b}}})$, Eq. (16), thereby violating the CHSH inequality under proper choice of the unit vectors $\hat{{\boldsymbol{a}}}=(\cos \,{\phi }_{a}\,\sin \,{\theta }_{a},\,\sin \,{\phi }_{a}\,\sin \,{\theta }_{a},\,\cos \,{\theta }_{a})$ and $\hat{{\boldsymbol{b}}}=(\cos \,{\phi }_{b}\,\sin \,{\theta }_{b},\,\sin \,{\phi }_{b}\,\sin \,{\theta }_{b},\,\cos \,{\theta }_{b})$ that fix the orientations of the measuring devices.

## Discussion

Bell violations prove wrong a conjunction that consists of three assumptions: realism, locality *and* Bell-type correlations. By rejecting last assumption and assuming correlations that are not of the Bell type, it becomes possible to construct a local-realistic model that leads to Bell violations. Bell’s theorem shows that quantum correlations cannot be of the Bell type. If realism and locality would imply Bell-type correlations, then Bell violations would prove wrong the conjunction consisting of realism and locality. However, as we have seen, realism and locality are compatible with correlations that lie outside the class of Bell-type correlations. They are inner-product-type correlations, having thus the same mathematical structure as the well known Pearson correlations of classical statistics. Quantum correlations stem from Born’s rule: *Tr*(*ρ*_*ϕ*_ *ρ*_*ψ*_), which is formally nothing but an Euclidean inner-product in disguise. As we have seen, under quite general assumptions, Gudder’s theorem leads to a signed-measure that is also an Euclidean inner product. By conveniently restricting the range of the function entering Gudder’s theorem, this function can be used as a probability measure. We can recover in this way Born’s rule, thereby showing that inner-product-type correlations are not exclusively related to quantum phenomena. We are entitled to use this type of correlations in the classical context, as much as we are entitled to use Born’s rule in the quantum context.

The model we have presented to exhibit Bell violations in a local-realistic context resorts to inner-product-type correlations, which are mathematically equivalent to Born’s rule. It is thus no surprise that we can obtain Bell violations. However, our model includes a prescription that is absent in the quantum formulation. This prescription refers to the action of the measuring device upon the system being measured. In our model, such an action is described by means of operators that act on Alice and Bob’s sites, see Eqs. (24, 25). In the context of Bell’s theorem, one postulates particular expressions for the correlations, 〈*AB*〉_*Bell*_ and 〈*AB*〉_*QM*_, see Eqs. (1, 2). These expressions do not derive from any physical model of the measuring process, but from considerations regarding probabilities alone, be they classical or quantal. Furthermore, inner-product-type correlations may involve vectors at two distant locations. This type of non-local expressions is not in conflict with local-realism and is not new, neither in physics nor in statistics, as exemplified by the Coulomb and Newtonian potentials on one side, and Pearson correlations on the other side.

Though in order to exhibit Bell violations we could have dealt only with the correlations entering Bell inequalities, we provided an *ad-hoc* model that reproduces all of Alice and Bob’s recordings in a Bell test. It has certainly not been our goal to replace the quantum description by a classical one, but to explore where the quantum-classical border lies. In order to claim that – in their very essence – all physical phenomena are quantum phenomena, we need a precise definition of what “quantum” means. Otherwise, such a claim seems to owe more to a doctrine than to science – or else to be, at the very least, meaningless.

### Appendix: Non-quantum entanglement and the inner-product probability measure

The quantum model used in Bell tests is based on two main assumptions: (1) Correlations are given by Born’s rule. (2) Physical systems can be produced in entangled states. Here we illustrate how these assumptions fit into a local-realistic description. To this end, let us consider the quantum correlation 〈*AB*〉_*QM*_ for the Bell state |Φ^+^〉 that we addressed before, see Eq. (18a). Last equation can be written in the form

A1 $$T{r}_{AB}[(\hat{{\boldsymbol{a}}}\cdot {{\boldsymbol{\sigma }}}^{A}\otimes \hat{{\boldsymbol{b}}}\cdot {{\boldsymbol{\sigma }}}^{B})|{{\rm{\Phi }}}^{+}\rangle \langle {{\rm{\Phi }}}^{+}|]={a}_{1}{b}_{1}-{a}_{2}{b}_{2}+{a}_{3}{b}_{3}\mathrm{.}$$

We notice that the right-hand side of the above equation can be straightforwardly obtained by writing

A2 $$|{{\rm{\Phi }}}^{+}\rangle \langle {{\rm{\Phi }}}^{+}|=\frac{1}{4}({{\mathbb{I}}}^{AB}+{\sigma }_{1}^{A}\otimes {\sigma }_{1}^{B}-{\sigma }_{2}^{A}\otimes {\sigma }_{2}^{B}+{\sigma }_{3}^{A}\otimes {\sigma }_{3}^{B})$$

and using *Trσ*_*i*_ = 0 together with the Pauli algebra: *σ*_*i*_ *σ*_*j*_ = *δ*_*ij*_ + *iε*_*ijk*_ *σ*_*k*_, where *i*, *j*, *k* ∈ {1, 2, 3} and *ε*_*ijk*_ means the completely antisymmetric Levi-Civita symbol. Eq. (A2) suggests the following approach.

We address two-level systems in the framework of a 4*D* vector space *V*_4_ with elements ${\boldsymbol{ {\mathcal R} }}\,:=({r}_{0},\,{r}_{1},\,{r}_{2},\,{r}_{3})$ and Euclidean inner product $\langle {\boldsymbol{ {\mathcal R} }},{\boldsymbol{ {\mathcal R} }}^{\prime} \rangle \,:={\sum }_{\mu =0}^{3}\,{r}_{\mu }{r}_{\mu }^{\text{'}}$. For the case of two two-level systems we consider the tensor product space ${V}^{AB}={V}_{4}^{A}\otimes {V}_{4}^{B}$ with orthonormal basis $\{{\hat{{\boldsymbol{e}}}}_{\mu }^{A}\otimes {\hat{{\boldsymbol{e}}}}_{\nu }^{B}\}$ and inner product $\langle {{\boldsymbol{ {\mathcal R} }}}_{A}\otimes {{\boldsymbol{S}}}_{B},{{\boldsymbol{ {\mathcal R} }}}_{A}^{\text{'}}\otimes {{\boldsymbol{S}}}_{B}^{\text{'}}\rangle \,:=$ $\langle {{\boldsymbol{ {\mathcal R} }}}_{A},{{\boldsymbol{ {\mathcal R} }}}_{A}^{\text{'}}\rangle \langle {{\boldsymbol{S}}}_{B},{{\boldsymbol{S}}}_{B}^{\text{'}}\rangle$, which is extended to all *V* ^*AB*^ by linearity. Gudder’s theorem leads us to connect inner-product measures with correlations. Having this in mind, we establish the following correspondences between quantum and classical quantities:

A3 $$\begin{array}{ccc}\hat{{\boldsymbol{a}}}\cdot {{\boldsymbol{\sigma }}}^{A} & \to  & {\bf{A}}={a}_{1}{\hat{{\boldsymbol{e}}}}_{1}^{A}+{a}_{2}{\hat{{\boldsymbol{e}}}}_{2}^{A}+{a}_{3}{\hat{{\boldsymbol{e}}}}_{3}^{A},\\ \hat{{\boldsymbol{b}}}\cdot {{\boldsymbol{\sigma }}}^{B} & \to  & {\boldsymbol{ {\mathcal B} }}={b}_{1}{\hat{{\boldsymbol{e}}}}_{1}^{B}+{b}_{2}{\hat{{\boldsymbol{e}}}}_{2}^{B}+{b}_{3}{\hat{{\boldsymbol{e}}}}_{3}^{B},\\ |{{\rm{\Phi }}}^{+}\rangle \langle {{\rm{\Phi }}}^{+}| & \to  & {\varphi }_{AB}^{+}={\hat{{\boldsymbol{e}}}}_{1}^{A}\otimes {\hat{{\boldsymbol{e}}}}_{1}^{B}-{\hat{{\boldsymbol{e}}}}_{2}^{A}\otimes {\hat{{\boldsymbol{e}}}}_{2}^{B}+{\hat{{\boldsymbol{e}}}}_{3}^{A}\otimes {\hat{{\boldsymbol{e}}}}_{3}^{B}\mathrm{.}\end{array}$$

Within the classical framework, we prescribe that the entangled state delivered by the source is represented by ${\varphi }_{AB}^{+}\in {V}^{AB}$, and that correlations between measurements performed by Alice and Bob along directions $\hat{{\boldsymbol{a}}}$ and $\hat{{\boldsymbol{b}}}$, respectively, are given by the inner product $\langle {\bf{A}}\otimes {\boldsymbol{ {\mathcal B} }},\,{\varphi }_{AB}^{+}\rangle$. This last prescription is, up to normalization, just an application of Gudder’s theorem to the case of the tensor-product space *V* ^*AB*^. From the definition of the inner product in *V* ^*AB*^, it follows that

A4 $$\langle {\bf{A}}\otimes {\boldsymbol{ {\mathcal B} }},{\varphi }_{AB}^{+}\rangle ={a}_{1}{b}_{1}-{a}_{2}{b}_{2}+{a}_{3}{b}_{3},$$

in accordance with Eq. (A1). As for the other three Bell states entering Eq. (18), we set

A5 $${\varphi }_{AB}^{-}=-{\hat{{\boldsymbol{e}}}}_{1}^{A}\otimes {\hat{{\boldsymbol{e}}}}_{1}^{B}+{\hat{{\boldsymbol{e}}}}_{2}^{A}\otimes {\hat{{\boldsymbol{e}}}}_{2}^{B}+{\hat{{\boldsymbol{e}}}}_{3}^{A}\otimes {\hat{{\boldsymbol{e}}}}_{3}^{B}$$

A6 $${{\boldsymbol{\psi }}}_{AB}^{+}={\hat{{\boldsymbol{e}}}}_{1}^{A}\otimes {\hat{{\boldsymbol{e}}}}_{1}^{B}+{\hat{{\boldsymbol{e}}}}_{2}^{A}\otimes {\hat{{\boldsymbol{e}}}}_{2}^{B}-{\hat{{\boldsymbol{e}}}}_{3}^{A}\otimes {\hat{{\boldsymbol{e}}}}_{3}^{B}$$

A7 $${{\boldsymbol{\psi }}}_{AB}^{-}=-{\hat{{\boldsymbol{e}}}}_{1}^{A}\otimes {\hat{{\boldsymbol{e}}}}_{1}^{B}-{\hat{{\boldsymbol{e}}}}_{2}^{A}\otimes {\hat{{\boldsymbol{e}}}}_{2}^{B}-{\hat{{\boldsymbol{e}}}}_{3}^{A}\otimes {\hat{{\boldsymbol{e}}}}_{3}^{B}$$

All the above vectors fit the definition of “entangled states”, in the sense that they cannot be written in the form ${\hat{{\boldsymbol{v}}}}^{A}\otimes {\hat{{\boldsymbol{w}}}}^{B}$. This definition of entanglement applies whenever we deal with tensor-product spaces and is not restricted to the quantum formalism alone^10,11^. We should stress that any non-local feature that we may ascribe to quantum Bell states can also be ascribed to classical, tensor-product states of the above type. Having reproduced quantum predictions with two models, one containing entangled states and the other not, we have shown that the inner-product probability measure is the primary ingredient leading to Bell violations. This ingredient may be consistently used within both a quantum description and a local-realistic description. The latter certainly includes classical optics. Here, fundamental features like coherence and polarization can be exhibited by means of different correlations, a prominent example of which is the spectral degree of coherence, $\eta ({P}_{1},\,{P}_{2},\,\omega )=\langle {\hat{{\boldsymbol{e}}}}^{\ast }({P}_{1},\,\omega )\cdot \hat{{\boldsymbol{e}}}({P}_{2},\,\omega )\rangle$, i.e., an inner-product probability measure^24^. The so-called, analytic signal $\hat{{\boldsymbol{e}}}({P}_{j},\,\omega )$ represents an electric field vector of frequency *ω* at location *P*_*j* = 1,2_. This vector has been unit-normalized with respect to ensemble average, which is the type of average that the angular brackets denote. Thus, for a transverse optical beam field propagating along the *z*-direction, we have, dropping explicit reference to the fixed frequency *ω*,

A8 $$\begin{array}{rcl}\langle {\hat{{\boldsymbol{e}}}}^{\ast }({P}_{1})\cdot \hat{{\boldsymbol{e}}}({P}_{2})\rangle  & \equiv  & \langle {e}_{x}^{\ast }({P}_{1}){e}_{x}({P}_{2})+{e}_{y}^{\ast }({P}_{1}){e}_{y}({P}_{2})\rangle \\  & := & \int {e}_{\mathrm{1,}x}^{\ast }{e}_{\mathrm{2,}x}p({e}_{\mathrm{1,}x},\,{e}_{\mathrm{2,}x}|{P}_{1},\,{P}_{2}){d}^{2}{e}_{\mathrm{1,}x}{d}^{2}{e}_{\mathrm{2,}x}\\  &  & +\int {e}_{\mathrm{1,}y}^{\ast }{e}_{\mathrm{2,}y}p({e}_{\mathrm{1,}y},\,{e}_{\mathrm{2,}y}|{P}_{1},\,{P}_{2}){d}^{2}{e}_{\mathrm{1,}y}{d}^{2}{e}_{\mathrm{2,}y}.\end{array}$$

The probability density functions entering the above definition, e.g., *p*(*e*_1,*x*_, *e*_2,*x*_|*P*_1_, *P*_2_), refer to different values of field components that are conditioned to be taken at two given locations, *P*_1_ and *P*_2_. Thus, *η*(*P*_1_, *P*_2_, *ω*) engages two degrees of freedom, field polarization and spatial location, while keeping fixed a third one: *ω* (alternatively, its Fourier-conjugate: time). Such a procedure can be variously used to exhibit different sorts of so-called cross-sector coherences, some of which might be initially hidden^3,7,12–16,37^.

Finally, notice that the spectral degree of coherence, *η*(*P*_1_, *P*_2_), is a quantity that can be seen, similarly to the quantum case, as involving two vectors, $\hat{{\boldsymbol{e}}}({P}_{1})$ and $\hat{{\boldsymbol{e}}}({P}_{2})$, which enter an expression that is not factorable with respect to, say, position: $\langle {e}_{x}^{\ast }({P}_{1}){e}_{x}({P}_{2})+{e}_{y}^{\ast }({P}_{1}){e}_{y}({P}_{2})\rangle \ne \langle f({P}_{1})g({P}_{2})\rangle$. Therefore, according to the general definition, *η*(*P*_1_, *P*_2_) represents an entangled observable. This observable, being given by Eq. (A8), has not the form of a Bell-type correlation: 〈*AB*〉_*Bell*_ = ∫*A*(*λ*)*B*(*λ*)*ρ*(*λ*)*dλ*. Hence, *η*(*P*_1_, *P*_2_) lies beyond the scope of Bell’s theorem and for this reason it may lead to Bell violations, in spite of being a full classical quantity.

## References

[1] Bell, J. S. *Speakable and unspeakable in quantum mechanics: collected papers in quantum philosophy*, Cambridge University Press, Cambridge (2004).

[2] Borges CVS, Hor-Meyll M, Huguenin JAO, Khoury AZ (2010). Bell-like inequality for the spin-orbit separability of a laser beam. Phys. Rev. A.

[3] Kagalwala KH, Di Giuseppe G, Abouraddy AF, Saleh EA (2013). Bell’s measure in classical optical coherence. Nat. Photonics.

[4] Aiello A, Töppel F, Marquardt C, Giacobino E, Leuchs G (2015). Quantum-like nonseparable structures in optical beams. New J. Phys..

[5] Qian X-F, Little B, Howell JC, Eberly JH (2015). Shifting the quantum-classical boundary: theory and experiment for statistically classical optical fields. Optica.

[6] McLaren M, Konrad T, Forbes A (2015). Measuring the nonseparability of vector vortex beams. Phys. Rev. A.

[7] Eberly JH (2015). Shimony-Wolf states and hidden coherences in classical light. Contemp. Phys..

[8] Balthazar WF (2016). Tripartite nonseparability in classical optics. Opt. Lett..

[9] Frustaglia D (2016). Classical physics and the bounds of quantum correlations. Phys. Rev. Lett..

[10] Spreeuw RJC (1998). A classical analogy of entanglement. Found. Phys..

[11] Eberly JH (2016). Quantum and classical optics – emerging links. Phys. Scr..

[12] Abouraddy AF, Yarnall T, Saleh BEA, Teich MC (2007). Violations of Bell’s inequality with continuous spatial variables. Phys. Rev. A.

[13] Svozilík J, Vallés A, Peřina J, Torres JP (2015). Revealing hidden coherence in partially coherent light. Phys. Rev. Lett.

[14] Kagalwala KH, Kondakci HE, Abouraddy AF, Saleh BEA (2015). Optical coherence matrix tomography. Sci. Rep..

[15] Eberly JH (2016). Correlation, coherence an context. Laser Phys..

[16] Qian X-F, Malhotra T, Vamivakas AN, Eberly JH (2016). Coherence constraints and the last hidden optical coherence. Phys. Rev. Lett..

[17] Spreeuw RJC (2001). Classical wave-optics analogy of quantum-information processing. Phys. Rev. A.

[18] Pinheiro da Silva B, Astigarreta Leal M, Souza CER, Galvão EF, Khoury AZ (2016). Spin-orbit laser mode transfer via classical analogue of quantum teleportation. J. Phys. B: At. Mol. Opt. Phys..

[19] Sandeau N, Akhouayri H, Matzkin A, Durt T (2016). Experimental violation of Tsirelson’s bound by Maxwell fields. Phys. Rev. A.

[20] Clauser JF, Shimony A (1978). Bell’s theorem: experimental tests and implications. Rep. Progr. Phys..

[21] Mermin ND (1993). Hidden variables and the two theorems of John Bell. Rev. Mod. Phys..

[22] Valdenebro AG (2002). Assumptions underlying Bell’s inequalities. Eur. J. Phys..

[23] Larsson J-Å (2014). Loopholes in Bell inequality tests of local realism. J. Phys. A: Math. Theor..

[24] Wolf, E. *Introduction to the Theory of Coherence and Polarization of Light*, Cambridge University Press, Cambridge (2007).

[25] Goodman, J. W. *Statistical Optics*, John Wiley & Sons, Inc., New York (2000).

[26] Gudder, S. P. *Stochastic Methods in Quantum Mechanics*, New York: North-Holland (1979).

[27] Cirelson BS (1980). Quantum generalizations of Bell’s inequality. Lett. Math. Phys..

[28] Gisin N (1991). Bell’s inequality holds for all non-product states. Phys. Lett. A.

[29] Kolgomorov, A. N. *Foundations of the theory of probability*, Chelsea Publishing Company, New York (1956).

[30] Gleason AM (1957). Measures on the closed subspaces of a Hilbert space. J. Math. Mech..

[31] Busch P (2003). Quantum States and Generalized Observables: A Simple Proof of Gleason’s Theorem. Phys. Rev. Lett..

[32] De Zela F (2016). Gleason-type theorem for projective measurements, including qubits: the Born rule beyond quantum physics. Found. Phys..

[33] Hall, M. J. W. Comment on “Gleason-type theorem for projective measurements, including qubits”, by F. De Zela, arXiv: 1611.00613v2 [quant-ph]

[34] De Zela, F. Reply on Gleason-type theorem for projective measurements, including qubits: the Born rule beyond quantum physics, by M. J. W. Hall, arXiv: 1611.09299v1 [quant-ph]

[35] Press, W. H., Flannery, B. P., Teukolsky, S. A. & Vetterling, W. T. *Numerical Recipes in FORTRAN: The Art of Scientific Computing*, 2nd ed., Cambridge, England: Cambridge University Press, pp. 630–633 (1992).

[36] Branciard C (2008). Testing quantum correlations versus single-particle properties within Leggett’s model and beyond. Nat. Phys..

[37] Eberly JH, Qian X-F, Vamivakas AN (2017). Polarization and coherence theorem. Optica.

